# Retrospective evaluation of transhepatic biliary stent placement in patients undergoing hepaticojejunostomy

**DOI:** 10.12669/pjms.41.11.12093

**Published:** 2025-11

**Authors:** Murat Akici, Emre Balli, Sezgin Yilmaz

**Affiliations:** 1 Murat Akici, Department of General Surgery, Afyonkarahisar Health Sciences University, Afyon, Turkey; 2 Emre Balli, Department of General Surgery, Afyonkarahisar Health Sciences University, Afyon, Turkey; 3 Sezgin Yilmaz, Department of General Surgery, Afyonkarahisar Health Sciences University, Afyon, Turkey

**Keywords:** Biliary, Injury, Transhepatic, Transanastomotic, Stent

## Abstract

**Objective:**

Cholecystectomy is one of the most common operations performed by surgeons all over the world. Bile duct injury (BDI) occurs in 0.2–0.6 % of cholecystectomies.

**Methodology:**

We aimed to investigate the effects of transhepatic transanastomotic stent use in patients who underwent Roux-en-Y hepaticojejunostomy due to iatrogenic bile duct injury. The study included 37 patients who underwent R&Y hepticojejunostomy anastomosis due to iatrogenic bile duct injury at the General Surgery Clinic of the Afyonkarahisar Health Sciences University Medical Faculty Hospital between January 2010 to July 2024.

**Results:**

A total of 37 hepaticojejunostomy patients were included in the study; 16 (%43.2) received transhepatic stent and 21 (%56.8) did not receive stent. No mortality occurred in any of the patients. It was observed that the rate of Bismuth Type-1 was statistically significantly higher in the group without stent placement and Bismuth Type-4 was statistically significantly higher in the group with stent placement (p<0.001). The mean timing time was 13.75±7.37 days in the group without stent placement and 4.00±3.30 days in the group with stent placement, which was significantly higher in the group without stent placement (p<0.001). No significant difference was observed between the groups in terms of complication development (p=0.933).

**Conclusions:**

As a result, although we prefer the use of transanastomotic stents in bile duct reconstruction after biliary injury in selected cases, we think that studies with larger series are needed.

## INTRODUCTION

Cholescystectomy is one of the most common operations performed by surgeons all over the world.^1^ Bile duct injury (BDI) occurs in 0.2–0.6 % of cholecystectomies.^2^ Laparoscopic surgery has increased these injuries in terms of both volume (0.4–0.6 %) as well as severity^3^ Less than half of these injuries are recognized during the operation; most of them are recognized in the early postoperative period.^4^ The two most frequent symptoms are bile leak and bile duct obstruction.^5^,^6^ Roux-en-Y hepaticojejunostomy (RHY) remains the best surgical alternative in patients where complete transection of the duct has occurred.^7^

Bile leakage after hepaticojejunostomy carries a high risk of prolonged hospitalization, biliary peritonitis, need for relaparotomy, and long-term development of biliary stenosis. The use of transanastomotic stents is controversial.^8^ Especially in patients whose bile ducts were torn during the anastomosis, or who suffered from bile leakage postoperatively, stents can play an important role in achieving complete healing of the anastomosis by maintaining continuity between the duct and jejunum, preserving the luminal space, and reducing the volume of bile leakage.^9^,^10^

## METHODOLOGY

In this study, we aimed to investigate the effects of transhepatic transanastomotic stent use in patients who underwent Roux-en-Y hepaticojejunostomy due to iatrogenic bile duct injury. The study included 37 patients who underwent R&Y hepticojejunostomy anastomosis due to iatrogenic bile duct injury at the General Surgery Clinic of the Afyonkarahisar Health Sciences University Medical Faculty Hospital between January 2010 to July 2024. Patients who underwent T-drainage or abdominal lavage alone due to iatrogenic bile duct injury were excluded from the study. The patients were divided into two groups: 16 patients (43.2%) who underwent transhepatic stent and 21 patients (56.8%) who did not undergo transhepatic stent. Patients were classified according to age, gender, injury class (Bismuth), time between injury and reconstruction (timing period), postoperative complications (cholangitis, abscess, fistula, bilioma, bleeding), length of hospital stay, and mortality.

### Ethical Approval:

We received approval from the Afyonkarahisar Health Sciences University Ethics Committee with Ref. number 2025/66.

### Operation Technique:

**Fig.1:**
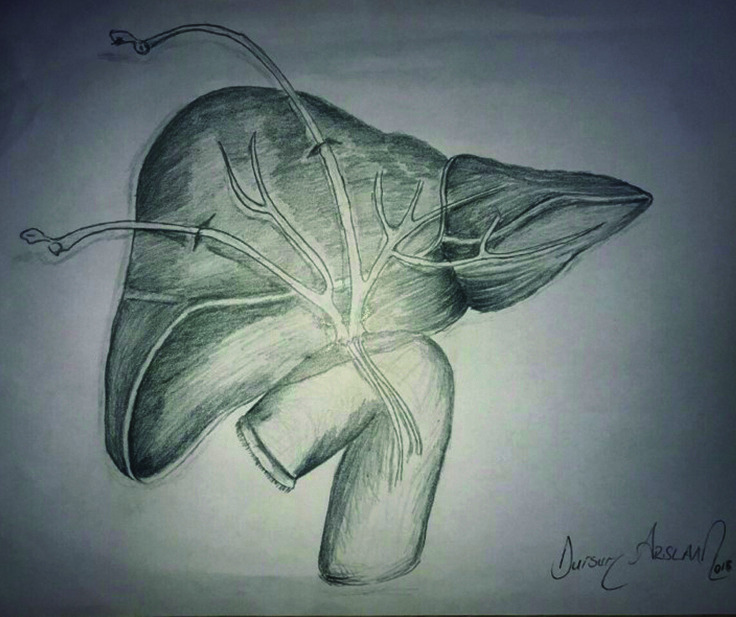
Transhepatic transanastomotic stent placement.

**Fig.2:**
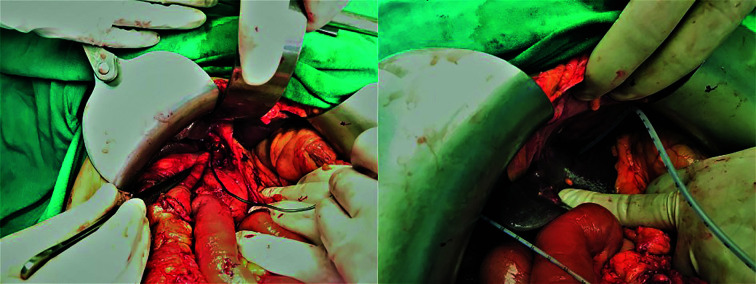
Intraoperative placement of a transhepatic catheter by sending a spark plug through the proximal common bile duct.

The proximal common bile duct was found and the distal jejunum was placed in the hepatic hilum in a retrocolic isoperistaltic manner. Spark plugs (3F) were removed by transhepatic passage through the right and left hepatic ducts separately. (Fig.1) After, the spark plug was pulled back so that the 1/0 silk seam was connected to the tip of the bobbin and the guide through the channel. 5F soft and perforated nutritional tube silk hemp was fixed. The silk suture was transhepatically pulled and the tube was placed and the skin was fixed. (Fig.2) The posterior wall of the anastomosis was made individually with 3/0 proline. The transhepatic stent from the right and left main hepatic duct was transanastomatically advanced in at least 5 cm through jejunum. The anastomotic front wall was completed with a single layer of 3/0 vycrille. An aspirative drain was placed near to the anastomosis and the operation was terminated.

### Statistical Analysis:

Statistical analysis of the study data was performed in a computer environment using IBM SPSS (Statistical Package for the Social Sciences) version 20.0 program. The conformity of the data to normal distribution was evaluated according to the Kolmogrov-Smirnov test, histogram graphics and Skewness-Kurtosis coefficients. In all tables, numerical variables were presented as mean ± standard deviation (SD) values, and categorical variables were presented as number (n) and percentage (%). In the comparison of binary groups; Student’s T test was applied for parameters showing normal distribution, and Mann-Whitney U test was applied for parameters showing abnormal distribution. The evaluation of multi-parameter cross tables was done with Chi-square test or Fisher Exact test. Logistic regression analysis method was used to analyze a data set with one or more independent variables determining a result. Hosmer-Lemeshow test was used for model fit. Results were considered statistically significant when p<0.05.

## RESULTS

A total of 37 hepaticojejunostomy patients were included in the study; 16 (%43.2) received transhepatic stent and 21 (%56.8) did not receive stent. No mortality occurred in any of the patients. No significant difference was found between the groups in terms of age and gender (p=0.914, p=0.893, respectively). In the group without transhepatic stent application; 10 (%62.5) patients had Bismuth Type-1, 5 patients had Type-2 (31.3%), and one patient had Type-3 (6.3%). Type-4 was not present in this group. In the group with transhepatic stent application; 1 (%4.8) patient had Type-1, 6 (%28.6) patients had Type-2, 8 (%38.1) patients had Type-3, and 6 (%28.6) patients had Type-4. It was observed that the rate of Type-1 was statistically significantly higher in the group without stent placement and Type-4 was statistically significantly higher in the group with stent placement (p<0.001).

The mean timing time was 13.75±7.37 days in the group without stent placement and 4.00±3.30 days in the group with stent placement, which was significantly higher in the group without stent placement (p<0.001). No significant difference was observed between the groups in terms of complication development (p=0.933). When the duration of hospitalization was evaluated; it was determined as 5.38±0.61 days in the group without stent placement and 4.81±0.68 days in the group with stent placement, which was statistically significantly higher in the group without stent placement (p=0.013) (Table-I).

**Table-I:** Patient data.

	Stent - N=16 Mean±SD (n %)	Stent+ N=21 Mean±SD (n %)	P
Age (year)	59.50±8.83	58.71±9.37	0.914
**Gender**			
*Female*	11 (68.8)	14 (66.7)	0.893
*Male*	5 (31.3)	7 (33.3)	
**Bismuth Classification**			
*1*	10 (62.5)	1 (4.8)	**<0.001**
*2*	5 (31.3)	6 (28.6)	
*3*	1 (6.3)	8 (38.1)	
*4*	0 (0)	6 (28.6)	
**Timing Time (day)**	13.75±7.37 13.50 (7-19)	4.00±3.30 4 (0-7)	**<0.001**
**Complications**			
*Yes*	4 (25)	5 (23.8)	0.933
*No*	12 (75)	16 (76.2)	
**Mortality**			
*Yes*	0 (0)	0 (0)	1.000
*No*	16 (100)	21 (100)	
Duration of hospitalizaion (day)	5.38±0.61	4.81±0.68	**0.013**

In our study, 9 out of 37 patients (24.3%) developed postoperative complications. 4 (44.4%) of the complications were in the group without transhepatic stent application and 5 (55.6%) in the group with transhepatic stent application. In the group without transhepatic stent application, 2 (50%) of the complications were fistula, 1 (25%) was cholangitis, and 1 (25%) was wound infection. In the group with transhepatic stent application, 2 (40%) of the complications were wound infection, 1 (20%) was cholangitis, 1 (20%) was renal failure, and 1 (20%) was abcess. The mean age of the patients without complications was 60.93±8.65, and the mean age of the patients with complications was 53.22±7.93, and it was statistically significantly lower in the group with complications than in the other group (p=0.024).

In the group with complications, 5 (55.6%) patients were female, and in the group without complications, 7 (25%) patients were female. Although the female gender ratio was higher in patients with complications, there was no statistically significant difference (p=0.088). There were no Bismuth Type-4 patients in the group with complications. Bismuth classification was similar between the groups (p=0.104). Timing time and hospital stay were similar between the groups (p=0.735, p=0.272, respectively). There was no significant difference in terms of stent placement rates in patients with and without complications (p=0.933) (Table-II).

**Table-II:** Comparison of demographic and clinical characteristics of the groups according to the development of complications.

	Complication- N=28 Mean±SD (n %)	Complication+ N=9 Mean±SD (n %)	P
Age (year)	60.93±8.65	53.22±7.93	**0.024**
**Gender**			
*Female*	7 (25)	5 (55.6)	0.088
*Male*	21 (75)	4 (44.4)	
**Bismuth Classification**			
*1*	10 (35.7)	1 (11.1)	0.104
*2*	7 (25)	4 (44.4)	
*3*	5 (17.9)	4 (44.4)	
*4*	6 (21.4)	0 (0)	
**Timing Time (day)**	7.54±6.10 6.50(3.25-10)	10.33±10.23 7(1.50-18)	0.735
**Group**			
*Stent –*	12 (42.9)	4 (44.4)	0.933
*Stent +*	16 (57.1)	5 (55.6)	
**Mortality**			1.000
*Yes*	0 (0)	0 (0)	
*No*	28 (100)	9 (100)	
Duration of hospitalizaion (day)	5.04±0.69	5.33±0.70	0.272

## DISCUSSION

Bile duct reconstruction after biliary injury is complex and requires a multidisciplinary approach. Biliary injuries are usually noticed in the early period and require intervention. In this period, there is an inflamed and ischemic choledoch. Anastomosis is difficult and the risk of complications is high. Guiliante F. et al.^11^ In their study on the effects of the timing of bile duct reconstruction, they revealed that the risk of cholangitis due to edema in the anastomosis, biliary leakage and long-term biliary stenosis in patients who underwent reconstruction, especially in the intraoperative and early postoperative periods, was higher than in patients who underwent late-term reconstruction. They attributed this to the higher risk of leakage due to ischemic and inflamed bile duct tissue in the early period. It was thought that the use of transanastomotic stents could prevent biliary leakage and stenosis by providing anastomotic patency and bile flow. While some centers routinely use stents, some centers avoid and oppose their use.^12^,^13^

In our study, we aimed to examine the effects of this use, as we prefer to use transanastomotic stents in selected cases in our clinic. In the group where transhepatic stent was used, the mean timing time was four days, while in the group where transhepatic stent was not used, we found a significant difference of 13.75 days, p<0.001. Again, in our study, we saw that we used stents in 14 out of 15 patients classified as bismuth 3 and 4. We do not routinely use stents in our clinic, but we can say that we prefer to use stents in patients with bismuth 3-4 and in whom we intervene in the early postoperative period. First of all, we can say that we use them because we encounter a more inflamed, ischemic and difficult to suture common bile duct in patients who have to intervene between the 1st and 5th days. Again, we prefer to use stents in high-placed injuries. Because we think that the common bile duct may be dissected more during the injury, disrupting its blood supply and increasing the risk of leakage.

If we examine the different opinions in the literature, Mercado MA et al^14^ In their comparative studies on stent use in patients undergoing reconstruction after biliary injury, they emphasized that stent use reduces complications, therefore they recommend stent use especially in cases of inflamed and ischemic common bile duct. Michelle Ong et al^15^ reported that there was no significant difference in complication rates in patients who used stents in hepaticojejunostomy anastomoses applied during liver transplantation compared to patients who did not use stents. Haberal M. et al^16^ emphasized that transhepatic stent use reduces complications in patients who underwent bile duct reconstruction during liver transplantation. Pamela L Valentino et al^17^ stated that routine stent use is not necessary in hepaticojejunostomy applications applied in pediatric liver transplantation patients despite the presence of a narrow common bile duct.

When we look at our study, we did not detect a significant difference in terms of complications between the two groups. However, biliary leakage was detected in two patients in the group where transanastomotic stent was not applied, while it was remarkable that no biliary leakage was observed in the group where transanastomotic stent was applied. Again, we found the hospital stay to be significantly lower in favor of the stent group. Although there was no difference in terms of complications, we attributed this situation to the positive effects of stent application on anastomosis safety thought by the surgical team and early discharge. When we look at the literature, there are studies on transanastomotic stent use after biliary injury in the late 90s and early 2000s. They emphasized the positive effects of stent use. However, the patient series in these studies are not large. In recent years, we have not observed any studies on stent use in bile duct reconstruction after biliary injury in the literature.

However, we see that there have been studies on liver transplantation applications regarding the use of transanastomotic stents in recent years. We see that there are different opinions in these studies. However, our study included an ischemic and inflamed hepaticojejunostomy anastomosis that was intervened in the early period after biliary injury. However, these liver transplantation studies included an elective, well-nourished and non-inflamed hepaticojejunostomy anastomosis. Therefore, we think that it is not right to evaluate the effects of stent use in liver transplantation applications comprehensively.

## CONCLUSION

As a result, although we prefer the use of transanastomotic stents in bile duct reconstruction after biliary injury in selected cases, we think that studies with larger series are needed.

### Author’s Contribution:

**MA:** Conceived, designed and did statistical analysis & editing of manuscript.

**MA, EB and SY:** Did data collection, manuscript writing, review and final approval of manuscript.

## References

[1] Shaikh AR, Shaikh AA, Abbasi M (2021). Short term outcomes of three dimensional versus two-dimensional laparoscopic cholecystectomy. Pak J Med Sci..

[2] Kong J, Malo JS, Hashem S, Mukharjee S, Lim J, Buell J (2025). Bile Duct Injury:A Novel Risk Stratification System for the Timing of Repair. Am Surg..

[3] Tantia O, Jain M, Khanna S, Sen B (2008). Iatrogenic biliary injury:13,305 cholecystectomies experienced by a single surgical team over more than 13 years. Surg Endosc..

[4] Fletcher R, Cortina CS, Kornfield H, Varelas A, Li R, Veenstra B (2020). Bile duct injuries:a contemporary survey of surgeon attitudes and experiences. Surg Endosc..

[5] Zarghami SY, Ghafoury R, Fakhar N, Afrashteh F, Tasa D, Hyder Z (2024). Four-Year Report of Iatrogenic Bile Duct Injury Repair from a Referral Hepatobiliary Center. Middle East J Dig. Dis.

[6] Lopez VL, Kuemmerli C, Maupoey J, Andujar RL, Lladó L, Mils K (2024). Textbook outcome in patients with biliary duct injury during cholecystectomy. Journal of Gastrointestinal Surgery.

[7] Zidan MHE, Eldeen MS, Ghazal AA, Refaie M (2024). Post-cholecystectomy bile duct injuries:a retrospective cohort study. BMC Surg..

[8] Suzuki H, Shimura T, Mochhida Y, Wada S, Araki K, Kubo N (2014). To Stent or Not to Stent Hepaticojejunostomy--Analysis of Risk Factors for Postoperative Bile Leaks and Surgical Complication. Hepatogastroenterology..

[9] Yadav TN, Pandit N, Deo KB, Lalijan Awale L, Neupane D, Shailesh Adhikary S (2024). Continuous versus interrupted anastomotic technique for the hepaticojejunostomy:a prospective cohort study Ann Med Surg.

[10] Ferrero A, Russolillo N, Vigano` L, Sgotto E, Lo Tesoriere R, Amisano M (2008). Safety of conservative management of bile leakage after hepatectomy with biliary reconstruction. J Gastrointest Surg..

[11] Giuliante F, Panettieri E, De Rose AM, Murazio M, Vellone M, Mele C (2023). Bile duct injury after cholecystectomy:timing of surgical repair should be based on clinical presentation. The experience of a tertiary referral center with Hepp-Couinaud hepatico-jejunostomy. Updates Surg..

[12] Salama IA, Shoreem HA, Saleh SM, Hegazy O, Housseni M, Abbasy M (2014). Iatrogenic Biliary Injuries:Multidisciplinary Management in a Major Tertiary Referral Center. HPB Surg. 2014.

[13] Wang GS, Yang Y, Jiang N, Fu BS, Li H, Li SH (2012). Routine use of a transanastomotic stent is unnecessary for hepatojejunostomy in liver transplantation. Chin Med J (Engl)..

[14] Mercado MA, Chan C,H (2002). Orozco To Stent or Not to Stent Bilioenteric Anastomosis After Iatrogenic Injury A Dilemma Not Answered?. Arch Surg.

[15] Ong M, Slater K, Hodgkinson P, Dunn N, Fawcett J (2018). To stent or not to stent:the use of transanastomotic biliary stents in liver transplantation and patient outcomes. ANZ J Surg..

[16] Haberal M, Karakayali H, Sevmis S, Boyvat F, Torgay A, Yilmaz U (2007). Intraoperative Transhepatic Biliary Catheter Insertion Technique for Biliary Reconstruction:Early Results. Transplant Proc..

[17] Valentino PL, Jonas MM, Lee CK, Kim HB, Vakili K, Elisofon SA (2016). Outcomes after discontinuation of routine use of transanastomotic biliary stents in pediatric liver transplantation at a single site. Pediatr transplant..

